# Mixed *Leptospira* Infections in a Diverse Reservoir Host Community, Madagascar, 2013–2015

**DOI:** 10.3201/eid2406.180035

**Published:** 2018-06

**Authors:** Mark Moseley, Soanandrasana Rahelinirina, Minoarisoa Rajerison, Benoit Garin, Stuart Piertney, Sandra Telfer

**Affiliations:** University of Aberdeen, Aberdeen, Scotland, UK (M. Moseley, S. Piertney, S. Telfer);; Institut Pasteur, Antananarivo, Madagascar (S. Rahelinirina, M. Rajerison, B. Garin);; Centre Hospitalier Universitaire, Les Abymes, Guadeloupe (B. Garin)

**Keywords:** spillover, co-infections, zoonoses, neglected tropical diseases, reservoir hosts, maintenance hosts, introduced species, invasive, public health, molecular epidemiology, disease ecology, bacteria, *Leptospira*, Madagascar

## Abstract

We identified mixed infections of pathogenic *Leptospira* in small mammals across a landscape-scale study area in Madagascar by using primers targeting different *Leptospira* spp. Using targeted primers increased prevalence estimates and evidence for transmission between endemic and invasive hosts. Future studies should assess rodentborne transmission of *Leptospira* to humans.

As a result of underreporting and lack of awareness, leptospirosis has been recognized as one of the world’s most neglected diseases (*1*). As is the case for other zoonotic pathogens, identifying key maintenance hosts and sources of human infection is essential for designing effective control strategies (*2*). However, leptospirosis epidemiology is complex; 10 pathogenic *Leptospira* species are phylogenetically delineated into 4 subgroups that differ in virulence and transmission (*3*), and multiple potential host species exist (*4*). In most studies, PCR protocols use primers targeting all pathogenic species, and the infecting *Leptospira* are identified on the basis of amplicon DNA sequence differences (*5*) or melt curve analyses (*6*). However, because of PCR primer biases or differences in infection intensities, such approaches probably underestimate mixed infections in areas with high *Leptospira* diversity.

Leptospirosis risk is high on islands in the western Indian Ocean; several *Leptospira* species on these islands are associated with disease (*7*). Studies in Madagascar have revealed acute cases of human leptospirosis and a seroprevalence of 3% (*7**–**9*). Studies of potential reservoirs in the region have revealed contrasting *Leptospira*–host associations. On Mayotte, an island neighboring Madagascar, 4 *Leptospira* species implicated in human disease (*L. interrogans* and *L. kirschneri* [taxonomic subgroup 1], *L. borgpetersenii* and *L. mayottensis *[taxonomic subgroup 2]) (*7*) have been detected in *Rattus rattus* rats (*6*), a highly successful invasive host introduced to the western Indian Ocean islands. However, *Tenrec ecaudatus *tenrecs, a mammal introduced from Madagascar, might also be a host of *L. mayottensis* (*10*). On Madagascar, only *L. interrogans* has been detected in invasive *Rattus* spp. rats (*11*), whereas *L. borgpetersenii*, *L. mayottensis*, and *L. kirschneri* have been detected in hosts endemic to Madagascar (*5*). In these studies, researchers did not attempt to identify mixed infections or sample invasive and endemic hosts from the same location, which would have been needed to fully assess *Leptospira* dynamics, spillover, and the role of hosts with widely different abundances and spatial distributions. Therefore, as part of a large landscape-scale study of *Leptospira* reservoirs in Madagascar, we developed quantitative PCRs (qPCRs) targeting individual *Leptospira* species and tested samples from small mammals to assess whether this approach changed our understanding of the reservoirs and spatial variation of risk.

## The Study

We conducted trapping and sample collection under permits issued by the Madagascar Ministry of Environment and Forests (no. 154/13/ MEF/SG/DGF/DCB.SAP/SCB; no. 312/13/MEF/SG/DGF/DCB.SAP/SCB; no. 178/14/MEF/SG/DGF/DCB.SAP/SCB). We conducted this study in accordance with Institut Pasteur animal use guidelines (https://www.pasteur.fr/en/file/2626/download? token=YgOq4QW7); the study was approved by a committee of the Institut Pasteur de Madagascar.

During 2013–2015, we sampled small mammal hosts at 11 sites in Moramanga District, eastern Madagascar. Two sites were within an uninhabited humid forest, and the remaining sites included areas of human habitation and heterogeneous land use. We identified host species on the basis of phenotypic characteristics, external measurements, and craniodental measurements (when appropriate) (*12*). We euthanized and dissected animals and stored kidneys in 95% ethanol.

We extracted DNA from 0.04 g of kidney tissue with DNeasy Blood and Tissue Kit (QIAGEN, Valencia, CA, USA) using the manufacturer’s instructions and an elution volume of 100 µL. We detected *Leptospira* with a TaqMan qPCR assay targeting the 16S rRNA gene (*13*) using the StepOne Real-Time PCR System (Life Technologies, Waltham, MA, USA). Any sample with a cycle threshold <36 in 1 assay or <40 in 2 replicate assays was classified as *Leptospira* positive.

Initial genotyping of positive samples was achieved by amplification and sequencing of ≈300 bp of the *lfb1 *gene on an Eco-Illumina qPCR System (Illumina Inc., San Diego, CA, USA) (*6*). To characterize mixed infections, we designed forward primers targeting the *lfb1* locus of 4 *Leptospira* species (*L. interrogans* 5′-CCTCTTACGCACAGATCRGTC-3′, *L. borgpetersenii* 5′-CCAACACTCCCTCCTCTATCAGC-3′, *L. mayottensis* 5′-CGCAGACTAGCAGCCCAACC-3′, and *L. kirschneri* 5′-GACCGCTTACGCACAGATCG-3′) and paired them with the standard *lfb1* reverse primer using the same thermal profile. After sequencing, we retested samples with redesigned primers targeting *Leptospira* spp. not previously identified and sequenced those products (GenBank accession nos. MG759567–664). Each assay included a negative control (sterile water) for every 4 samples and a positive control. We purified PCR products using the QIAquick PCR Purification Kit (QIAGEN) and sent them to Eurofins Genomics GmbH (Ebersburg, Germany) for sequencing. We calculated prevalence and logit CIs using the binom package in R version 3.2.2 (https://cran.r-project.org/package=binom).

We captured 2,847 small mammals across 11 sites; 5 invasive species (*R. rattus* and* R. norvegicus* rats, *Mus musculus* mice, and* Suncus murinus* and* S. etruscus* shrews) accounted for 93% (2,653/2,847) of the captures. Of these, we captured *R. rattus* rats most frequently (n = 2,312) and at all sites, including forest sites. Although we found endemic hosts at all sites, 56% (107/190) were captured at forest sites. We tested 723 captured animals (43–102 animals/site) for *Leptospira*. We tested all endemic host samples and a subset of introduced host samples for each site. Overall prevalence of infection was 26%, ranging from 11% in *Microgale* spp. tenrecs to 48% in *M. musculus* mice (Table).

**Table:** Prevalence of *Leptospira* infection in small mammal hosts, Madagascar, 2013–2015

Host type and species	No. positive/no. tested	Prevalence, % (95% CI)
Endemic		
* Microgale* spp. tenrecs*	12/108	11 (6–19)
* Eliurus* spp. rats*	6/24	25 (12–46)
* Hemicentetes semispinosus* tenrec	6/29	21 (10–39)
Invasive		
* Rattus rattus *rat	80/347	23 (19–28)
* Suncus murinus* shrew	16/60	27 (17–39)
* R. norvegicus* rat	17/36	47 (32–63)
* Mus musculus* mouse	57/119	48 (39–57)
Total	194/723	27 (24–30)

*Endemic *Microgale* tenrecs and *Eliurus* rats were analyzed at the genus level because these genera were composed of a large number of species.

We genotyped 93 *Leptospira*-positive samples; the prevalence of mixed infections was 19% (95% CI 13%–29%). This value is still likely an underestimate, considering that cross-amplification of *Leptospira* species within the same taxonomic subgroup occurred. Mixed infections comprised *L. interrogans* and either *L. borgpetersenii* (n = 14) or *L. mayottensis* (n = 3); 1 animal was infected with all 3 species. All mixed infections were detected in rodents (order Rodentia): 78% (14/18) in *R. rattus* rats, 11% (2/18) in *R. norvegicus* rats, and the remaining 2 in endemic *Nesomys rufus* mice and *Eliurus minor* rats. After characterizing mixed infections, the proportion of *R. rattus* rats infected with *L. borgpetersenii* nearly doubled (Figure), and the number of *L. mayottensis*–infected *R. rattus* rats equaled the number of *L. mayottensis*–infected endemic hosts (n = 4). All of the *L. mayottensis*–infected *R. rattus* rats were captured at sites with human habitation; 75% (3/4) of *L. mayottensis*–infected endemic hosts were captured at forest sites. The *L. interrogans* *lfb1* genotype most commonly identified was identical to the *lfb1* sequence obtained from a human with a case of leptospirosis contracted in Madagascar (*9*).

**Figure:**
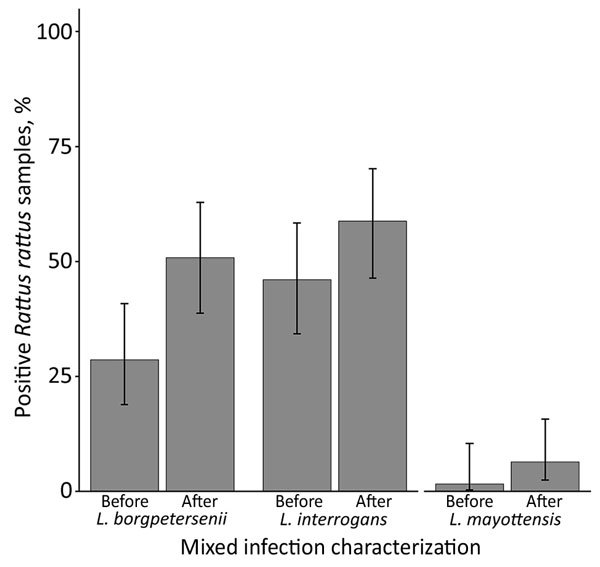
Proportion of *Leptospira*-positive *Rattus rattus* rat samples (n = 63) infected with *L. borgpetersenii*, *L. interrogans*, or *L. mayottensis* before and after characterizing mixed infections, Madagascar, 2013–2015. We initially genotyped *R. rattus* rat samples positive for *Leptospira* 16S rRNA by sequencing ≈300 bp of the *lfb1* gene using standard primers and thermal profile (*6*). To characterize mixed infections, we used forward primers targeting the *lfb1* locus of the different *Leptospira* species and the standard reverse primer and thermal profile. Mixed infections result in the sum of proportions exceeding 100% after characterization. Error bars represent 95% CIs.

## Conclusions

We present definitive molecular evidence that small mammal hosts carry mixed infections of pathogenic *Leptospira* spp. The characterization of mixed infections and testing of sympatric endemic and invasive reservoir hosts has altered our understanding of leptospirosis epidemiology. Previously, only *L. interrogans* was detected in *Rattus* spp. rats in Madagascar (*11*). We show that, when mixed infections are characterized, the prevalence of *L. borgpetersenii* and *L. interrogans* in *R. rattus* rats is similar. Similar to findings from Mayotte (*14*), *R. rattus* rats are a potential source of human infection for 3 of the 4 *Leptospira* species present in Madagascar. Because of the high abundance and widespread distribution of these rats, they could act as a key reservoir for *Leptospira*, including for *L. mayottensis*, which might occur as spillover infections from endemic species.

The high prevalence of mixed *Leptospira* infections also provides a potential explanation for the genetic and serologic diversity of pathogenic *Leptospira* in the region (*5*,*7*), considering horizontal genetic transfer has been implicated in *Leptospira* evolution, including evolution of the locus responsible for serologic classification (*rfb*) (*3*). Further work is needed to better characterize the evolutionary and landscape-scale epidemiologic consequences of mixed infections. Moreover, the prevalence of infection and the identification of an *lfb1* genotype in *Rattus* spp. rats identical to that in a human case (*9*) suggests that rodentborne transmission of *Leptospira* might be an underreported health problem in Madagascar. Studies on human exposure are urgently needed.

## References

[1] Hartskeerl RA, Collares-Pereira M, Ellis WA. Emergence, control and re-emerging leptospirosis: dynamics of infection in the changing world. Clin Microbiol Infect. 2011;17:494–501. 10.1111/j.1469-0691.2011.03474.x 21414083

[2] Viana M, Mancy R, Biek R, Cleaveland S, Cross PC, Lloyd-Smith JO, et al. Assembling evidence for identifying reservoirs of infection. Trends Ecol Evol. 2014;29:270–9. 10.1016/j.tree.2014.03.002 24726345 PMC4007595

[3] Picardeau M. Virulence of the zoonotic agent of leptospirosis: still terra incognita? Nat Rev Microbiol. 2017;15:297–307. 10.1038/nrmicro.2017.5 28260786

[4] Bharti AR, Nally JE, Ricaldi JN, Matthias MA, Diaz MM, Lovett MA, et al.; Peru-United States Leptospirosis Consortium. Leptospirosis: a zoonotic disease of global importance. Lancet Infect Dis. 2003;3:757–71. 10.1016/S1473-3099(03)00830-2 14652202

[5] Dietrich M, Wilkinson DA, Soarimalala V, Goodman SM, Dellagi K, Tortosa P. Diversification of an emerging pathogen in a biodiversity hotspot: *Leptospira* in endemic small mammals of Madagascar. Mol Ecol. 2014;23:2783–96. 10.1111/mec.12777 24784171

[6] Merien F, Portnoi D, Bourhy P, Charavay F, Berlioz-Arthaud A, Baranton G. A rapid and quantitative method for the detection of *Leptospira* species in human leptospirosis. FEMS Microbiol Lett. 2005;249:139–47. 10.1016/j.femsle.2005.06.011 16006065

[7] Bourhy P, Collet L, Lernout T, Zinini F, Hartskeerl RA, van der Linden H, et al. Human *leptospira* isolates circulating in Mayotte (Indian Ocean) have unique serological and molecular features. J Clin Microbiol. 2012;50:307–11. 10.1128/JCM.05931-11 22162544 PMC3264139

[8] Ratsitorahina M, Rahelinirina S, Michault A, Rajerison M, Rajatonirina S, Richard V; 2011 Surveillance Workshop group. Has Madagascar lost its exceptional leptospirosis free-like status? PLoS One. 2015;10:e0122683. 10.1371/journal.pone.0122683 25874381 PMC4396993

[9] Pagès F, Kuli B, Moiton M-PP, Goarant C, Jaffar-Bandjee M-CC. Leptospirosis after a stay in Madagascar. J Travel Med. 2015;22:136–9. 10.1111/jtm.12163 25319525

[10] Lagadec E, Gomard Y, Le Minter G, Cordonin C, Cardinale E, Ramasindrazana B, et al. Identification of *Tenrec ecaudatus*, a wild mammal introduced to Mayotte Island, as a reservoir of the newly identified human pathogenic *Leptospira mayottensis.* PLoS Negl Trop Dis. 2016;10:e0004933. 10.1371/journal.pntd.0004933 27574792 PMC5004980

[11] Rahelinirina S, Léon A, Harstskeerl RA, Sertour N, Ahmed A, Raharimanana C, et al. First isolation and direct evidence for the existence of large small-mammal reservoirs of *Leptospira* sp. in Madagascar. PLoS One. 2010;5:e14111. 10.1371/journal.pone.0014111 21124843 PMC2991340

[12] Soarimalala V, Goodman SM. Les petits mammifères de Madagascar. Antananarivo (Madagascar): Association Vahatra; 2011.

[13] Smythe LD, Smith IL, Smith GA, Dohnt MF, Symonds ML, Barnett LJ, et al. A quantitative PCR (TaqMan) assay for pathogenic *Leptospira* spp. BMC Infect Dis. 2002;2:13. 10.1186/1471-2334-2-13 12100734 PMC117785

[14] Desvars A, Naze F, Vourc’h G, Cardinale E, Picardeau M, Michault A, et al. Similarities in *Leptospira* serogroup and species distribution in animals and humans in the Indian ocean island of Mayotte. Am J Trop Med Hyg. 2012;87:134–40. 10.4269/ajtmh.2012.12-0102 22764304 PMC3391038

